# Human plasma-like medium (HPLM) induces *Cryptococcus neoformans in vivo* cell morphologies

**DOI:** 10.1128/msphere.00281-24

**Published:** 2024-05-21

**Authors:** Eduardo G. Mozo, Orlando Ross, Raif Yuecel, Ivy M. Dambuza, Liliane Mukaremera

**Affiliations:** 1 Medical Research Council Centre for Medical Mycology, University of Exeter, Exeter, United Kingdom; 2 Faculty of Health and Life Sciences, Exeter Centre for Cytomics, The Bioeconomy Centre, University of Exeter, Exeter, United Kingdom; University of Michigan Michigan Medicine, Ann Arbor, Michigan, USA

**Keywords:** *Cryptococcus neoformans*, cell morphology, HPLM, titan cells

## Abstract

The fungal pathogen *Cryptococcus neoformans (C. neoformans*) forms yeast cells of different sizes and morphological characteristics during infection. These features are usually not seen in standard laboratory *in vitro* conditions. Here, we describe *in vivo* cell morphologies when *C. neoformans* is grown in human plasma-like medium at 37°C, 5% CO_2_. We observed mixed-size populations of cells less than 1 µm up to 16.8 µm in cell diameter, increased capsule size, high chitin, and DNA content in larger cells. Our findings show that serum is not required for human plasma-like medium (HPLM)-induced *C. neoformans* cellular heterogeneity. Thus, this new method offers an opportunity to investigate factors of *C. neoformans* that mediate pathogenesis or host–pathogen interactions in a physiologically relevant setting.

**IMPORTANCE:**

We provide a description of new *in vitro* culture condition using the human plasma-like medium that supports the formation of the full range of *in vivo* cell morphologies of *C. neoformans*.

## OBSERVATION

The fungal pathogen, *Cryptococcus neoformans*, forms yeast cells of different sizes and morphological characteristics *in vivo*. Some of these dynamic cell populations include typical sized yeasts (5–7 μm), enlarged cells referred to as titan cells (> 10 µm), and small cells (less than 5 µm) that include micro cells, drop cells, seed cells and titanides (1–4). In contrast to *in vivo* conditions where significant size heterogeneity exists, *C. neoformans* grown under nutrient rich conditions *in vitro* predominantly produce typical sized yeast cells with a uniform morphology. Most *in vitro* studies on *C. neoformans* have used these homogenous cell populations, which, although convenient, do not represent the broad range of cellular morphologies present during infection, and therefore may lead to inaccurate conclusions about factors that mediate *C. neoformans* host–pathogen interactions and pathogenesis.

In 2018, three protocols for the production of titan cells *in vitro* were described (4–6). The factors found to induce the formation of titan cells include (i) low nutrients, hypoxia, low pH, and continuous shaking (5); (ii) low nutrients, neutral pH, and presence of serum and azide in static conditions with a CO_2_-enriched atmosphere (6); and (iii) low nutrients and presence of serum in static conditions with a CO_2_-enriched atmosphere (4). Although there are differences between these three protocols, there are also common themes such as growth under low nutrients, oxygen limitation, and low cell density conditions.

**Fig 1:**
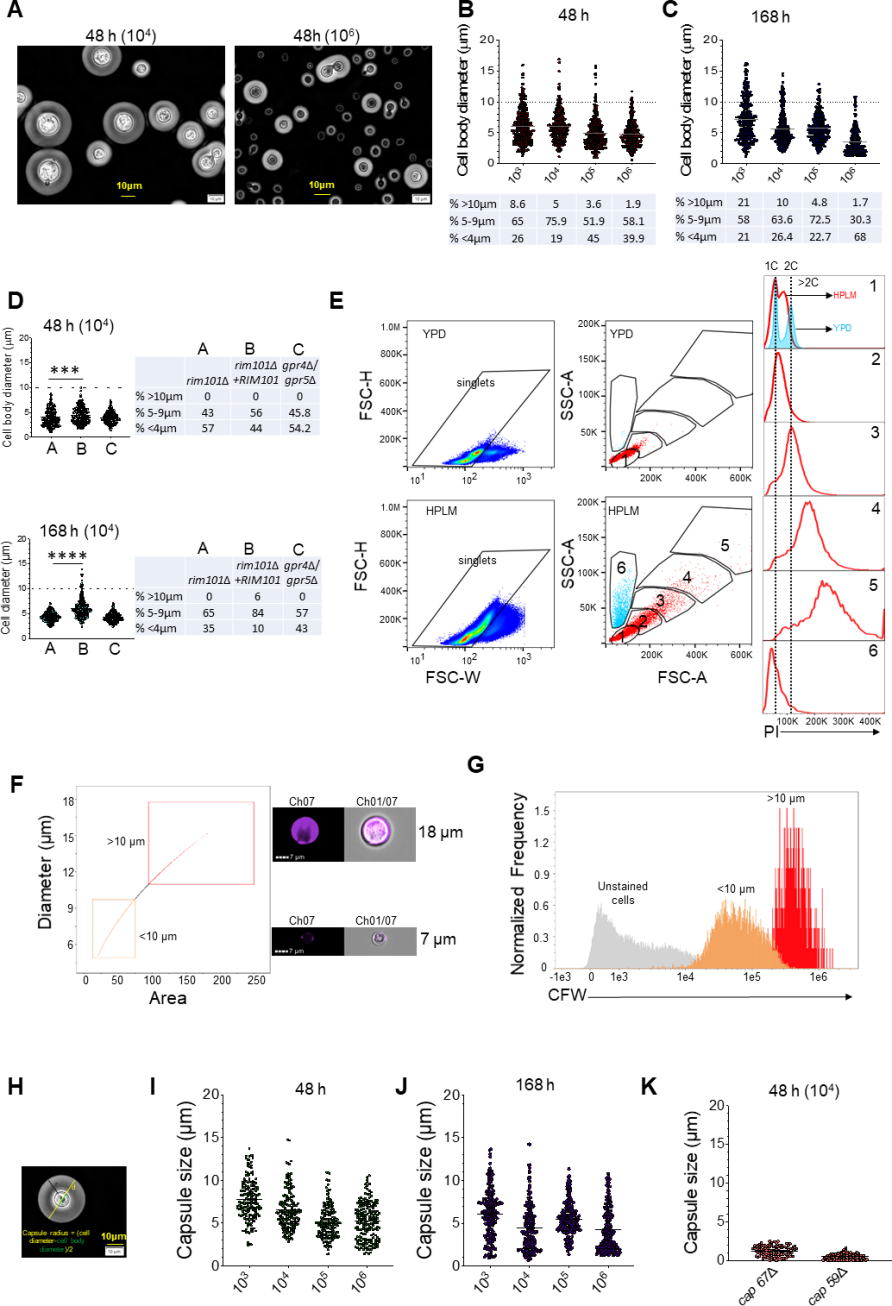
*Cryptococcus neoformans* forms *in vivo*-like morphologies in HPLM at 37°C, 5% CO_2_. *C. neoformans* H99 and various mutant strains were grown overnight in YPD at 30°C with shaking (200 rpm), then washed twice with sterile water, and counted with hemocytometer. Various inoculum concentrations were added to six well plates containing HPLM, and incubated at 37°C, 5% CO_2_ for 48 h or 168 h. After incubation, *C. neoformans* cells were analyzed for their cell body (diameter), capsule sizes, DNA content, and chitin content. (**A) ***C. neoformans* cells were fixed with formaldehyde, suspended in India ink, and imaged on an Olympus CKX53 microscope. (**B and C) **Cell diameters were measured using ImageJ at 48 h (**B**) and 168 h (**C**). Data presented are representative of three biological replicates with at least 300 cells. (**D) **Cell size of mutants with defects in titan cell formation. 10^4^ cells/ml was cultured in HPLM as described above. Data presented are representative of four biological replicates with at least 300 cells measured. ***P < 0.001, ****P < 0.0001 (Mann–Whitney test). (**E) ***C. neoformans* H99 cells were grown in YPD or HPLM for 48 h, washed in cold sterile water, fixed with 80% methanol, and stained with 50 µg/mL PI for 30 min. DNA content was measured using flow cytometry for populations 1–6. (**F and G) **To measure the chitin content, 10^6^ cells/mL were stained with 25 µg/mL Calcofluor white for 5 minutes, washed, and then analyzed by imaging flow cytometry. Calcofluor white (for chitin content) stained cells were analyzed by imaging flow cytometer. Data presented are from 8,544 singlets. (**H) **Diagram of capsule radius measurement. (**I-J) **Capsule thickness/radius of *C. neoformans* H99 after 48 h (**I**) and 168 h (**J**) of incubation in HPLM. Data presented are representative of four biological replicates with at least 160 cells measured. (**K) **Capsule thickness of mutants with defects in capsule formation. Cells (10^4^ cells/mL) were incubated in HPLM, and data presented are representative of three biological replicates with at least 100 cells measured. The grey line in figures represents the median. The dotted line at Y-axis represents the 10 µm cut-off, and the percentage of all cell subpopulations are presented in the tables below the graphs (**B and C**) or next to the graphs (**D**). DNA: deoxyribonucleic acid, PI: propidium iodide, YPD: yeast extract-peptone-dextrose, HPLM: human plasma-like medium.

Here, we describe new *in vitro* conditions that induce the formation of cell morphologies, which mimic the diversity of cell morphologies observed *in vivo*. To induce the formation of *in vivo* like *Cryptococcus* cell morphologies, the wild-type H99 strain was used. Overnight cultures in nutrient-rich yeast extract-peptone-dextrose (YPD) were inoculated into the “human plasma-like medium” (HPLM) and then incubated at 37°C, 5% CO_2_. HPLM is a relatively new culture medium that was developed using salts and polar metabolites similar to those present in the adult human plasma (7). We observed that *Cryptococcus* can grow and multiply in HPLM medium. Microscopic observation of cells grown in HPLM shows a population of cells with sizes ranging from small cells of less than 1 µm to large cells up to 16.8 µm in cell diameter (Fig. 1A through C). Using previously described cell body size (diameter) measurements, *C. neoformans* cells were divided into three sub-populations: titan cells (>10 µm), normal-sized yeasts (5–9 μm), and smaller cells (<4 µm). We observed a mixture of all three cell sub-populations in our cultures (Fig. 1A through C). Similar to previous observations, low cell density induced more cells with a diameter of more than 10 microns when compared to high cell density inocula at 48 and 168 h of incubation (Fig. 1B and C, respectively). We also tested strains known to have defects in titan cell formation *in vivo* and *in vitro*, the *rim101*Δ and *gpr4*Δ/*gpr5*Δ mutants (8). Our results show that these two mutants had defects in large cell formation (>10 µm) in HPLM (Fig. 1D). *In vivo* titan cells are polyploid and have an increased chitin content. Therefore, we used flow cytometry to examine the DNA and cell-wall chitin content after staining with propidium iodide (PI) and calcofluor white (CFW), respectively. We analyzed *Cryptococcus* grown in HPLM and nutrient rich YPD (control). Typical 5–7 μm cells in YPD had 1C and 2C DNA contents (population 1, Fig. 1E). Similar sized cells (population 1) from HPLM-grown cells also had a majority of 1C and, to a lesser extent, 2C DNA content. Notably, a smaller size population of cells was also observed that had varying side scatter properties, suggestive of cellular diversity (population 6, Fig. 1E). This unique population observed in HPLM was predominantly comprised of cells with 1C DNA content. Consistent with published reports (5, 9), the large cells (populations 4 and 5) had higher DNA content that was >2C (Fig. 1E). Similarly, the large cells (> 10 µm) had increased cell-wall chitin content compared to the typical sized and small cells in HPLM (Fig. 1F and G) as previously described for *in vivo* titan cells (5, 10).

In addition to various cell sizes, cells grown in HPLM had a large capsule (Fig. 1A and H). In HPLM, the capsule radius varied between 1.6 and 13.5 µm (median 6.3) in titan cells and 0.6 to 12.8 µm (median 5.4) in non-titan cells (Fig. 1I and J). This is bigger than previously described *in vitro* cells that had a median capsule radius of 4.8 µm in titan and 2.7 µm in typical cells *in vitro* (5). Interestingly, some HPLM-grown cells displayed capsule sizes up to 13.5 µm, which is close to sizes observed for *in vivo* titan cells (14.8 µm) (5). Interestingly, when cell body diameter was plotted against capsule size, we observed that even non-titan cells have larger capsules while some of the titan cells had smaller capsules, further demonstrating the phenotypic heterogeneity of cells grown in HPLM (Fig. S1). Moreover, in head-to-head comparison between HPLM and RPMI medium, Dulbecco’s modified Eagle medium (DMEM), or YPD, only RPMI induced larger cells (>10 µm) similar to HPLM but remarkably only HPLM induced larger capsules (Fig. S2). Mutants with known defects in capsule formation (*cap59*Δ and *cap67*Δ) (11, 12) showed small to no capsule formation in HPLM (Fig. 1K). These findings show that the large cells formed in HPLM possess the characteristics of *in vivo* titan cells.

Small *C. neoformans* cells were previously characterized depending on various morphological factors, and have been designated by multiple names including micro cells (~1 µm) (13), titanides (oval, metabolically active with a thin cell wall) (4), drop cells (metabolically inactive with a thick cell wall) (14), or seed cells (similar to titanides but have increased mannose exposure and are seen in cultures devoid of titan cells) (3). It is not known whether all these different small cells are present at the same time during infection. Based on the varying side scatter properties of these small cells, we hypothesize that the small cell population generated in HPLM contains a mixture of small cell morphologies.

**Fig 2:**
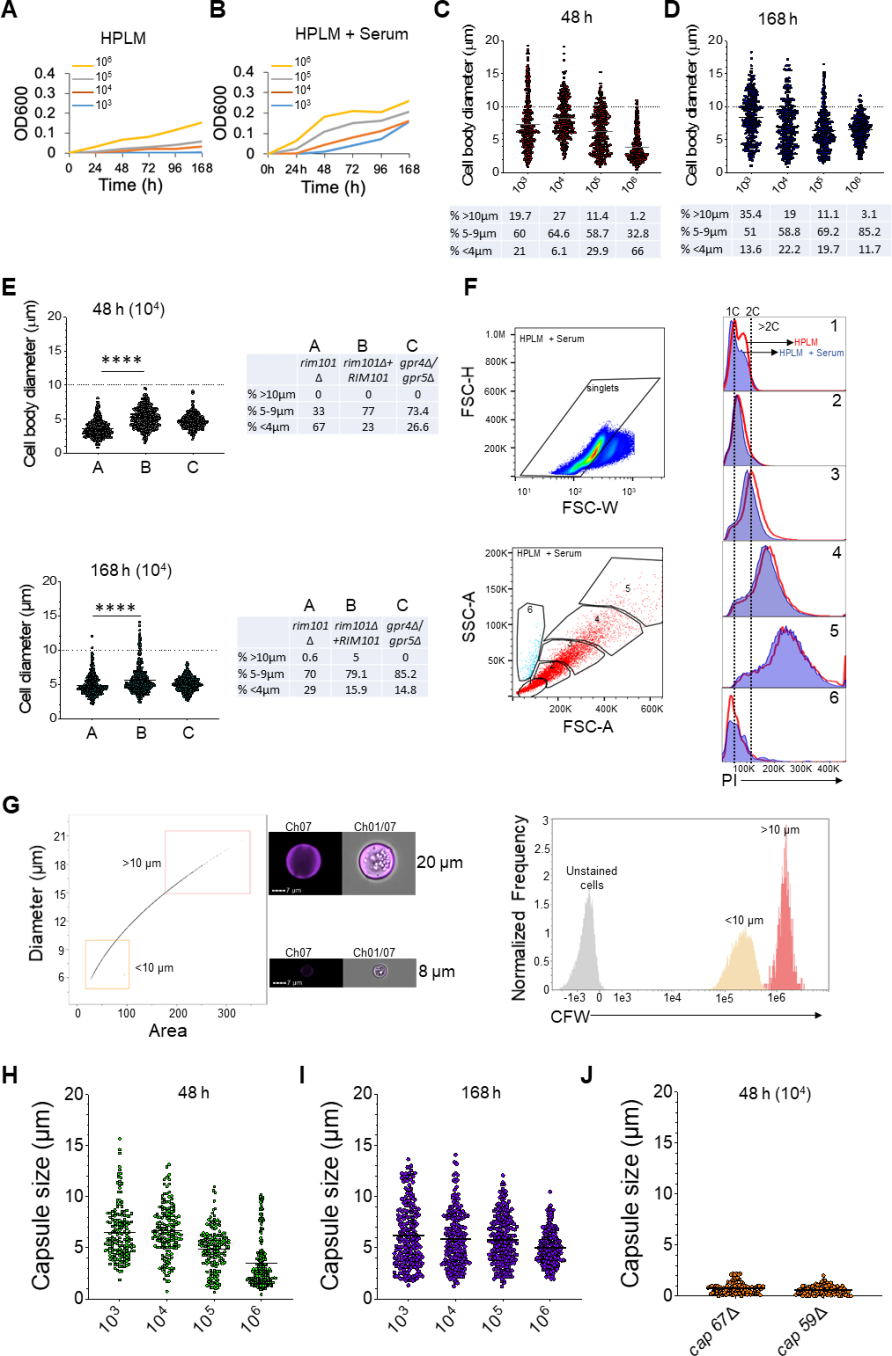
Serum is dispensable for HPLM-induced *Cryptococcus neoformans* cellular heterogeneity. *C. neoformans* H99 and various mutant strains were grown overnight in YPD at 30°C with shaking (200 rpm), then washed twice with sterile water, and counted with hemocytometer. Various inoculum concentrations were added to six well plates containing HPLM supplemented with 10% FBS, and incubated at 37°C, 5% CO_2_ for 48 h or 168 h. After incubation, *C. neoformans* cells were analyzed for their cell body (diameter), capsule sizes, DNA content, and chitin content. (**A and B) **Growth curve of *C. neoformans* H99 in HPLM (**A**) and HPLM supplemented with FBS (**B**). Optical density (OD600) was read at 0, 24, 48, 72, 96, and 168 h of incubation. (**C and D) **Cell body sizes of *C. neoformans* H99 in HPLM supplemented with FBS at 48 h (**C**) and 168 h (**D**) of incubation. Data presented are representative of three biological replicates with at least 300 cells. (**E) **Cell body sizes of mutants with defects in titan cell formation at 48 h of incubation. Data presented are representative of four biological replicates with at least 300 cells measured. ****P < 0.0001 (Mann–Whitney test) (**F) **DNA content of H99 cells grown in HPLM (red line, overlaid Fig. 1E) and HPLM supplemented with 10% FBS (blue background). (**G) **To measure chitin content, 10^6^ cells/mL was stained with 25 µg/mL Calcofluor white for 5 minutes, washed, and then analyzed by imaging flow cytometry. (**H and I) **Capsule thickness (radius) of H99 cells grown in HPLM and 10%FBS at 48 h (**H**) and 168 h of incubation (**I**). Data presented are representative of four biological replicates with at least 160 cells measured. (**J) **Capsule thickness of mutants with defect in the capsule formation grown in HPLM supplemented with FBS at 48 h incubation. Data presented are representative of three biological replicates with at least 100 cells measured. The grey line in figures represents the median. The dotted line at Y-axis represents the 10 µm cutoff, and the percentage of all cell subpopulations is presented in tables below the graphs (**C and D**) or next to the graphs (**E**). DNA: deoxyribonucleic acid, FBS: fetal bovine serum, PI: propidium iodide, HPLM: human plasma-like medium.

Serum has been previously described to be an inducer of titan cell formation *in vitro* (4, 6). We supplemented the HPLM medium with serum to determine whether it would enhance the production of titan cells. *C. neoformans* grew slowly in HLPM alone (Fig. 2A) contrary to the fast growth observed in HPLM supplemented with 10% fetal bovine serum (FBS) (Fig. 2B). There was also an increase in the number of titan cells in the presence of FBS, both at 48 h (Fig. 2C) and 168 h of incubation (Fig. 2D). For example, titan cells (>10 µm) represented 35.4% of the whole population at 168 h in the presence of serum, while they were 21% in HPLM alone when the initial inoculum was 10^3^ cells (Fig. 1C and 2D). Mutants with defects in titan cell formation behaved similarly in the presence and absence of serum (Fig. 1D and 2E). DNA content was also similar in both HPLM and HPLM supplemented with 10% FBS (Fig. 2F, overlaid on HLPM alone). In addition, the capsule sizes were comparable in HLPM or HPLM supplemented with serum (Fig. 2H through J). These data show that the presence of serum provided a boost in *C. neoformans*-titan cell formation, but HPLM alone was sufficient to induce cellular heterogeneity similar to that observed *in vivo*. Statistical analyses comparing the cell body and capsules sizes of cells grown in HPLM with and without serum are presented in the supplemental material (Tables S1 and S2).

Collectively, the results presented here show that HPLM, a medium that more closely resembles the human plasma, which was incubated at 37°C with 5% CO_2_, can be used to induce the diversity of *C. neoformans* cell morphologies observed *in vivo*. HPLM has been used for immunological studies where it induced a different transcriptional response in human primary T-cells and improved their activation after antigen stimulation (15). To our knowledge, HPLM has not been used to grow fungal pathogens and we show, for the first time, that *C. neoformans* grow and differentiate into *in vivo* cell morphologies in this medium. Thus, HPLM is a great option to use in experiments investigating *C. neoformans* pathogenesis, specifically host-*Cryptococcus* interactions, as it can be used to culture both the fungus and the host immune cells. For example, HPLM is the optimal growth medium when co-culturing *C. neoformans* with human primary T-cells *in vitro*. Future work should focus on identifying how this media influences *Cryptococcus* gene expression that leads to the formation of different cell morphologies in *C. neoformans* and other members of the *Cryptococcus* species complex.

## SUPPLEMENTAL MATERIAL

Supplemental material msphere.00281-24-s0001.docx Supplemental tables and figures.

ASM does not own the copyrights to Supplemental Material that may be linked to, or accessed through, an article. The authors have granted ASM a non-exclusive, world-wide license to publish the Supplemental Material files. Please contact the corresponding author directly for reuse.
