# Supplementary material for: Human plasma-like medium (HPLM) induces Cryptococcus neoformans in vivo cell morphologies
Source: mSphere. 2024 May 21;9(6):e00281-24. doi: 10.1128/msphere.00281-24 (PMC11332328; doi:10.1128/msphere.00281-24)
Supplement: Supplemental material — Supplemental tables and figures. [file msphere.00281-24-s0001.docx]

**Table S1 Statistical analysis for cell body sizes of *C. neoformans* grown in HPLM with and without fetal bovine serum**

|  |  | **48 h** | | | **168 h** | | |
| --- | --- | --- | --- | --- | --- | --- | --- |
|  |  | **HPLM** | **HPLM +Serum** | **HPLM vs HPLM +Serum** | **HPLM** | **HPLM +Serum** | **HPLM vs HPLM +Serum** |
| **Strain** | **Cell Density** | **Median** | **Median** | **P-value** | **Median** | **Median** | **P-value** |
| H99 | 10^3^ | 5.585 | 6.403 | 0.0001 | 6.771 | 8.442 | < 0.0001 |
|  | 10^4^ | 5.732 | 7.846 | < 0.0001 | 4.931 | 7.24 | < 0.0001 |
|  | 10^5^ | 4.444 | 5.825 | < 0.0001 | 5.461 | 5.796 | 0.0017 |
|  | 10^6^ | 4.43 | 3.003 | < 0.0001 | 2.917 | 6.716 | < 0.0001 |
| *rim101*Δ | 10^4^ | 3.665 | 3.414 | 0.0033 | 4.287 | 4.544 | 0.0001 |
| *rim101*Δ+*RIM101* | 10^4^ | 4.204 | 5.104 | <0.0001 | 5.597 | 5.137 | 0.0002 |
| *gpr4*Δ/*gpr5*Δ | 10^4^ | 3.864 | 4.595 | <0.0001 | 4.19 | 4.957 | <0.0001 |

Mann Whitney test was used to compare sizes of *C. neoformans* cells grown in HPLM and HPLM supplemented with 10% fetal bovine serum.

**Table S2 Statistical analysis for capsule sizes of *C. neoformans* grown in HPLM with and without fetal bovine serum**

|  |  | **48 h** | | | **168 h** | | |
| --- | --- | --- | --- | --- | --- | --- | --- |
|  |  | **HPLM** | **HPLM +Serum** | **HPLM vs HPLM +Serum** | **HPLM** | **HPLM +Serum** | **HPLM vs HPLM +Serum** |
| **Strain** | **Cell Density** | **Median** | **Median** | **P-value** | **Median** | **Median** | **P-value** |
| H99 | 10^3^ | 7.404 | 5.905 | <0.0001 | 6.145 | 5.688 | 0.8975 |
|  | 10^4^ | 6.173 | 6.439 | 0.2901 | 3.923 | 5.548 | <0.0001 |
|  | 10^5^ | 5.008 | 5.087 | 0.4118 | 5.347 | 5.647 | 0.257 |
|  | 10^6^ | 5.35 | 2.559 | <0.0001 | 3.74 | 4.801 | <0.0001 |
| *cap67*Δ | 10^4^ | 1.279 | 0.72 | <0.0001 |  |  |  |
| *cap59*Δ | 10^4^ | 4.204 | 0.56 | 0.1576 |  |  |  |

Mann Whitney test was used to compare the capsule radius of *C. neoformans* cells grown in HPLM and HPLM supplemented with 10% fetal bovine serum.

**Figure S1**

**
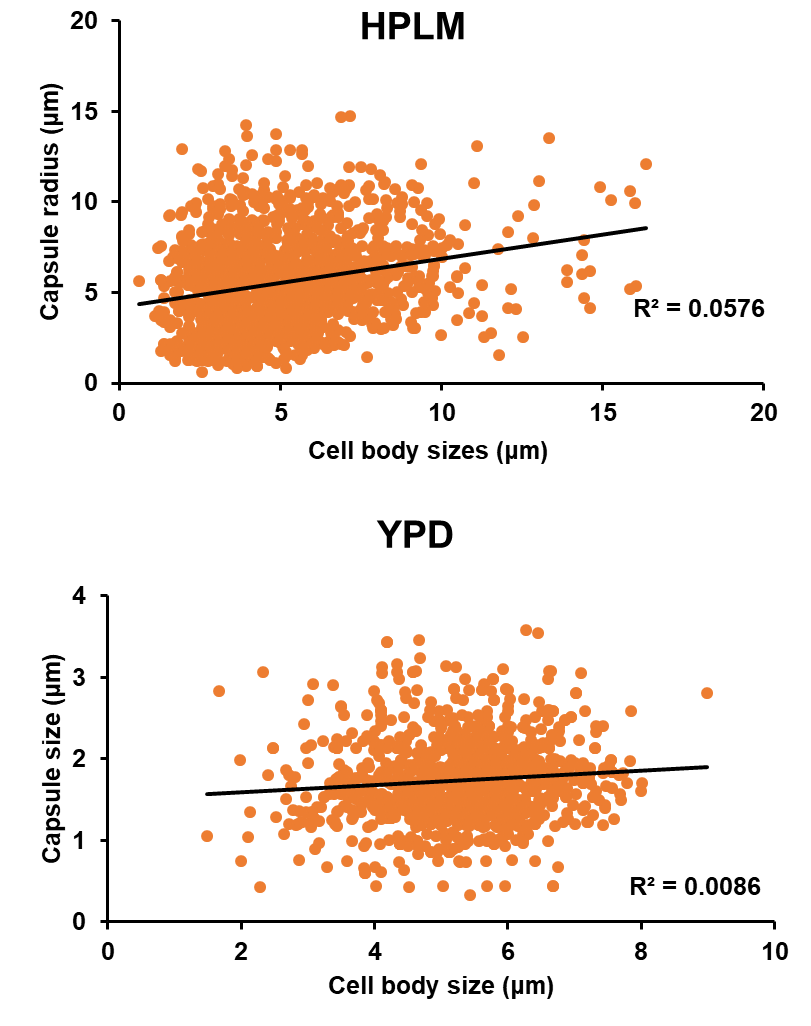
**

**Figure S1 Correlation between cell body sizes and capsule radius of *C. neoformans* grown in HPLM.** *C. neoformans* H99 strain was grown overnight in YPD at 30°C with shaking (200 rpm), then washed twice with sterile water and counted with hemocytometer. Aliquots of each initial inoculum (10^3^, 10^4^, 10^5^, 10^6^ cells/ml) were added to 6-well plates (2 ml per well) containing HPLM media, and incubated at 37°C, 5% CO_2_ for 48 hours. Control cultures in YPD (similar inocula as in HPLM) were incubated at 30°C for 48 hours without shaking. After incubation, *C. neoformans* cells were analysed for their cell body (diameter), and capsule sizes (radius). *C. neoformans* cells were fixed with formaldehyde, suspended in India ink and imaged on an Olympus CKX53 microscope. Cell diameters and capsule radius were measured using ImageJ. Data presented are representative of 3 biological replicates and data from all inocula were combined for the correlation analysis. At least 1000 cells were used in the correlation analysis.

**Figure S2**

**
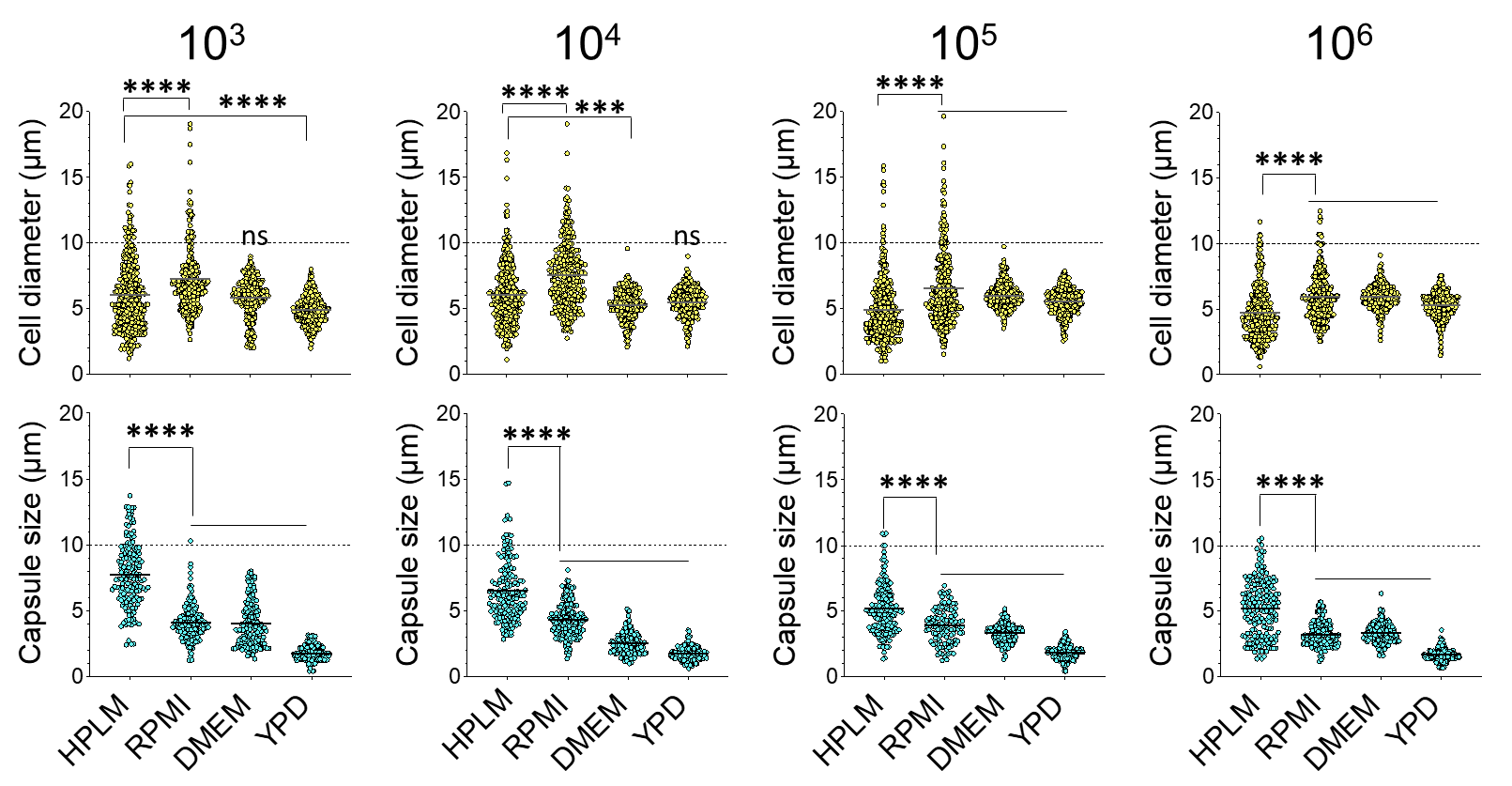
**

**Figure S2 Cell body and capsule sizes of *C. neoformans* grown in HPLM, RPMI, DMEM and YPD.** *C. neoformans* H99 strain was grown overnight in YPD at 30°C with shaking (200rpm), then washed twice with sterile water and counted with hemocytometer. Various inoculum concentrations were added to 6 well plates containing HPLM, RPMI and DMEM and incubated at 37°C, 5% CO_2_ for 48 h. Cultures in YPD media were incubated at 30°C for 48 h without shaking. After the incubation, *C. neoformans* cells were fixed with formaldehyde, suspended in India ink and imaged on an Olympus CKX53 microscope. Cell diameters and capsule radius were measured using ImageJ. Data presented are representative of 3 biological replicates with at least 300 cells measured. The grey line in figures represents the median. The dotted line at Y-axis represents the 10-micron cut-off. Mann Whitney test was used to compare C. neoformans cell body and capsule sizes when grown in HPLM, RPMI, DMEM and YPD. *** P<0.001, **** P<0.0001, ns: differences not statistically significant. DMEM: Dulbecco's Modified Eagle Medium, RPMI: Roswell Park Memorial Institute, YPD: yeast extract-peptone-dextrose.
